# Annual incidence of osteoporotic hip fractures in Iran: a systematic review and meta-analysis

**DOI:** 10.1186/s12877-021-02603-1

**Published:** 2021-11-30

**Authors:** Kiarash Tanha, Noushin Fahimfar, Shahrzad Nematollahi, Sayed Mahmoud Sajjadi-Jazi, Safoora Gharibzadeh, Mahnaz Sanjari, Kazem Khalagi, Fatemeh Hajivalizedeh, Alireza Raeisi, Bagher Larijani, Afshin Ostovar

**Affiliations:** 1 Department of Biostatistics, School of Public Health, Iran University of Medical Sciences, Tehran, Iran; 2 Osteoporosis Research Center, Endocrinology and Metabolism Clinical Sciences Institute, Tehran University of Medical Sciences, Tehran, Iran; 3 Men’s Health and Reproductive Health Research Center, Shahid Beheshti University of Medical Sciences, Tehran, Iran; 4 Cell Therapy and Regenerative Medicine Research Center, Endocrinology and Metabolism Molecular-Cellular Sciences Institute, Tehran University of Medical Sciences, Tehran, Iran; 5 Endocrinology and Metabolism Research Center, Endocrinology and Metabolism Clinical Sciences Institute, Tehran University of Medical Sciences, Tehran, Iran; 6 Department of Epidemiology and Biostatistics, Pasteur Institute of Iran, Tehran, Iran; 7 Noncommunicable Disease Center, Ministry of Health, Tehran, Iran; 8 School of Medicine, Shiraz University of Medical Sciences, Shiraz, Iran

**Keywords:** Osteoporosis, Osteoporotic fractures, Incidence, Iran, Meta-analysis

## Abstract

**Background:**

Osteoporosis (OP) is progressively becoming a global concern with the aging of the world’s populations. Osteoporotic fractures are associated with significantly increased mortality rates and a financial burden to health systems. This Meta-analysis aims to estimate the annual incidence of osteoporotic fractures in Iran.

**Methods:**

A comprehensive systematic literature search was performed through Medline (PubMed), Embase, Scopus, Web of Science, and Google Scholar to identify studies which contain an investigation of the incidence of osteoporotic fractures in Iran up to December 3rd 2020, with no time and language restriction. For the risk of bias assessments of studies, the Joanna Briggs Institute (JBI) critical appraisal checklist for studies reporting prevalence data was used. The pooled estimation of the incidence of osteoporotic fractures in population aged≥50 years was calculated using random-effects meta-analysis, and the heterogeneity of included studies was quantified with the I^2^ statistic.

**Results:**

In all, 6708 papers were initially retrieved from the electronic databases, among which seven studies were included in the meta-analysis. The pooled standardized annual cumulative incidence of hip fractures was estimated as 138.26 (95% CI: 98.71–193.65) per 100,000 population and 157.52 (95% CI: 124.29–199.64) per 100,000 population in men and women, respectively.

**Conclusion:**

This study showed a high incidence rate of osteoporotic hip fractures in Iran. Early detection and treatment of individuals with higher risks of primary fragility fractures at primary health care as well as implementing fracture liaison services to prevent secondary fractures are highly recommended. The results suffer from the following limitations: first, a low number of studies that were eligible for inclusion; second, the lack of population-based studies; and presence of highly heterogeneous studies despite the use of a random effect model.

**Supplementary Information:**

The online version contains supplementary material available at 10.1186/s12877-021-02603-1.

## Background

Osteoporosis (OP), defined by low bone mass and microarchitectural deterioration of bone tissue, is a common issue for global health [1–3] and is gradually becoming a global concern with the aging of the world’s populations. It was reported that 200 million women are affected by osteoporosis worldwide [4]. The health burden of osteoporosis has been authenticated by health authorities leading to reports and guidelines on prevention and management in developed countries. In Iran, the prevalence of spinal osteoporosis was estimated as 11.0% in men and 19% in post-menopausal women [5]. Osteoporosis is getting to be a health issue as Iran affected by aging. Recent projections indicate that approximately 5 million people will have osteoporosis by 2050 [6].

As a silent disease, it is mostly undiagnosed until a fracture happens. Osteoporotic fractures, all fractures from low energy trauma that would not rise to fracture in healthy people, increase with age after 50 years [7]. Annually, osteoporosis causes about 9 million fractures [8]. Currently, 1 in 3 women and 1 in 5 men over the age of 50 will sustain osteoporotic fractures in their lifetime. Every fracture increases the probability of another impending one in the future [9]. Osteoporotic fractures cause an annual global loss of 5.8 million healthy life years to disability [10].

In Iran, there were 50,000 osteoporotic fractures in 2010, and it is estimated to increase to 62,000 fractures by 2020 [11]. Osteoporotic fractures are associated with significantly increased mortality rates, decreased quality of life, and a financial burden to health systems [12]. Although all bones are susceptible to osteoporotic fractures, the most common osteoporotic fractures occur in the hip, spine, and forearm [13]. Total Disability-Adjusted Life Year (DALY) was 36,026 years for hip, spine, and forearm fractures [14].

Having precise information about the incidence rate of osteoporotic fractures is critical for large-scale strategic planning to prevent fractures and their subsequent burden. Considering the need for a comprehensive study in Iran, conducting a systematic review and meta-analysis study can provide relevant, precise, and valid results. This study aims to estimate the annual incidence rate of osteoporotic fractures in Iran.

## Materials and methods

### Eligibility criteria

All the original articles which contain an investigation of the incidence of osteoporotic fractures in Iran were considered regardless of study setting (national surveys, hospital-based or community-based). No restriction of study time, language, gender of participants, or age cut-offs was considered. In the case of lack of information for the type of fractures, osteoporotic fractures were defined as the fractures associated with low bone mass or fractures after the age of 50 years resulted by a minor trauma.

### Search strategy

A comprehensive search adhering to the Preferred Reporting Items for Systematic reviews and Meta-Analyses (PRISMA) guidelines was performed using the databases of Medline (via PubMed), Embase (via Elsevier), Scopus, Web of Science (core collection; Clarivate), and Google Scholar to identify the English articles published until December 3rd, 2020 (Supplementary File 1). Persian databases including SID, IranDoc, Magiran, and Civilica were searched using the same keywords in Farsi. Additionally, the reference lists of retrieved articles were hand-searched for additional references.

### Study selection

**Fig. 1:**
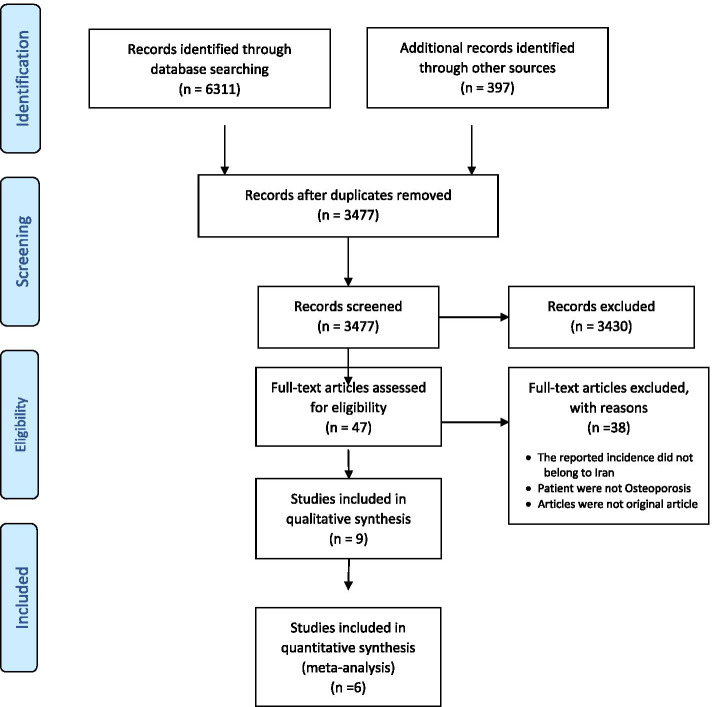
PRISMA flowchart of study selection

After identifying a total of 6708 articles and removing the duplicates, 3477 records were reviewed by title and abstract by two investigators (K.T and S.N) independently. In case of any disagreement, it was resolved upon a third researcher’s discussion and judgment (N.F). Then, the full texts of 47 articles were reviewed to appraise the eligibility criteria, and the articles of interest were selected. The selection process of the articles is presented in Fig. 1.

In some studies, the crude rates, or standardized rates based on the 2000 U.S. population were only reported. To achieve a comparable index, standardized rates were calculated for all papers using article information and the 2000 U.S. population.

Some articles had used hospital-based data which were a mix of osteoporotic and non-osteoporotic cases. Since this study aimed to estimate the rate of osteoporotic fractures, we have excluded the data of traffic injuries and extracted the relevant data by carefully studying the tables and text. In the case of lack of information for the type of fractures, osteoporotic fractures were defined as the fractures associated with low bone mass or fractures after the age of 50 years resulted by a minor trauma.

### Data extraction

A pre-designed data sheet was completed for each of the included studies. Data extraction from each included paper was performed by two independent authors (K.T and S.N) based on the author’s name, publication year, journal name, study population, city of the study population, sex, sample size, and incidence rate.

### Risk of bias assessment

As the risk of bias assessment should be considered based on the type of primary studies, the Joanna Briggs Institute (JBI) critical appraisal checklist for studies reporting prevalence data was used to assess each selected study’s quality [15]. Two investigators (K.T and S.N) independently assessed the methodological quality of primary studies. In case of uncertainty or disagreement between reviewers, an independent investigator was consulted to reach a consensus (N.F).

### Statistical analysis

To provide comparable results, the age-standardized annual cumulative incidence of hip fractures was calculated. Most included studies were used the 2000 U.S population as a reference population to calculate the age-standardized annual incidence of hip fractures; Using this reference population, direct age adjustment was applied to adjust the reported incidence rates through age-categories. Finally, for conducting the meta-analysis, overall incidence rate of hip fracture has been calculated using adjusted rates in all age categories for each study and was included in the analysis.

The data were analyzed using STATA, version 13.0 (STATA Corporation, College Station, TX, USA). Metaprop package was used to estimate the pooled incidence of osteoporotic fractures and the associated 95% CI. This package uses double arcsine transformations to stabilize the variance in the meta-analyses. The statistical heterogeneity between the studies was assessed using Cochran’s Q test (from chi-square) and the I^2^ statistic, which was able to measure the inconsistency across the results of the studies and describe the proportion of the total variations based on their estimates due to the presence of heterogeneity rather than sampling errors. A random-effects model using DerSimonian and Laird method was used if heterogeneity was observed (the I^2^ values > 50). Applying separate analysis by sex, a meta-regression on study characteristics (e.g., year of publication, province, and study design) was planned to identify the reasonable sources of heterogeneity. Publication bias was assessed in more than three studies using Egger’s test.

## Results

In all, 6708 papers were initially retrieved from the electronic databases, among which seven studies were included in the meta-analysis (Fig. 1), containing 4260 persons. Table 1 presents the main characteristics of the selected studies.

**Table 1:** Main characteristics of the selected studies

ID	Author name	Year	Province	Mean age	Number of hip fractures	Measure	Standardized	Crude Rate (per 100,000 person)	Standardized rate (per 100,000 person)
1	Ghafoori et.al.	2014	Tehran	54.2 ± 11.5	23	Incidence rate	–	252 in pooled sex per 100,000 person-year	–
2	Moayyeri et.al.	2004	Multi-Province	–	450	Cumulative incidence	U.S. population 2000	84.37 in men	113.03 in men^b^
								103.24 in women	137.83 in women^b^
3	Abolhassani et.al. ^a^	2006	Multi-Province	59.9 ± 20.7 in men 70.2 ± 15.3 in women	450	Cumulative incidence	U.S. population 2000	84.37 in men	113.03 in men^b^
								103.24 in women	137.83 in women^b^
4	Moayyeri et.al. ^a^	2006	Multi-Province	–	450	Cumulative incidence	U.S. population 2000	84.37 in men and	113.165 in men
								103.24 in women	138.31 in women
5	Maharlouei et.al.	2017	Fars	74.9 ± 11.9 in men 76.7 ± 10.4 in women	605	Cumulative incidence	U.S. population 2000	–	66.51 in men
									92.37 in women
6	Soveid et.al	2005	Fars	74.2 ± 9.2 in men 74.29 ± 9.2 in women	1833	Cumulative incidence	U.S. population 1989	71.28 in Men	128.2 in men
								112.72 in Women	182.7 in women
7	Valizadeh et.al.	2008	Zanjan	72.0 ± 10.6 in men 76.0 ± 11.4 in women	244	Cumulative incidence	U.S. population 2000	190.9 in Men	206.5 in men
								160.3 in Women	214.8 in women
8	Asgarzadeh et.al.	2008	East Azarbaijan	75.0	779	Cumulative incidence	U.S. population 2000	176 in Men	200.07 in men^b^
								174 in Women	190 in women^b^
9	Beyranvand et.al.	2009	Kermanshah	71.4 ± 11.9 in men 73.5 ± 9.9 in women	161	Cumulative incidence	U.S. population 2000	125.13 in Men	165.77 in men ^b^
								109.57 in Women	153.51 in women^b^

^a^ The Iranian Multicenter Study on Accidental Injuries (IMSAI) dataset was used in these two studies as the same as Moayyeri et.al. [16]. Consequently, these studies have not been included in the meta-analysis

^b^ Standardized rates were calculated using article information

**Fig. 2:**
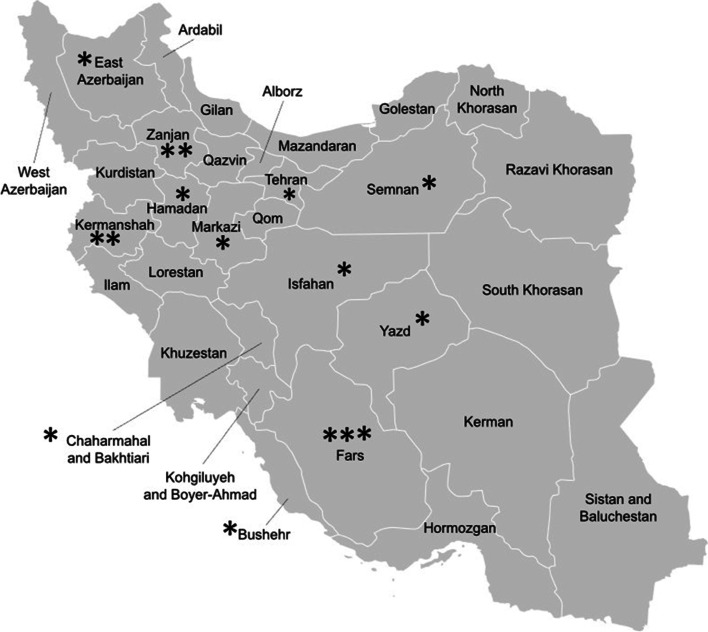
Geographic dispersion pattern of included studies on map (each * represent the number of studies reported in the province) dssdd

All the included studies had been published after 2003. Only one paper addressed both major osteoporotic and hip fractures while the rest targeted the hip fractures. The number of patients ranged between 161 and 1833 individuals. One of the studies used data derived from the Iranian Multicenter Study on Accidental Injuries (IMSAI) which is a national population-based study conducted in 9 different provinces [17]. Other studies were performed in a single province which collected data may be obtained from government or private hospitals. Fig. 2 shows the geographic dispersion pattern of included studies on the map. The Person-year incidence rate of osteoporotic fractures was reported in only one study [18]. The annual incidence rate of major osteoporotic fractures and hip fractures were reported as 613 and 252 per 100,000 person-year, respectively.

**Fig. 3:**
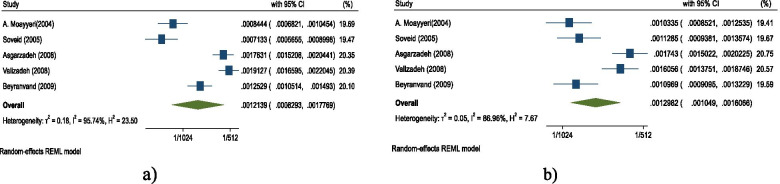
Hip fractures meta-analysis. **a** Pooled annual cumulative incidence in men **b** Pooled annual cumulative incidence in women

**Fig. 4:**
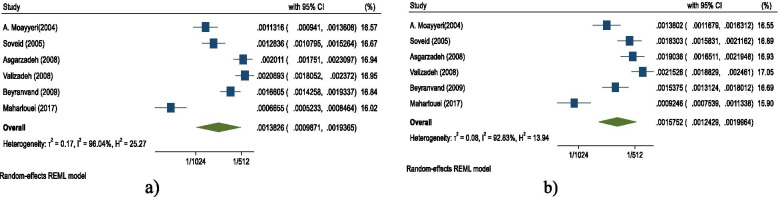
Age-standardized annual cumulative incidence. **a** Pooled rate in men **b** Pooled rate in women

Data was provided only for hip fractures in the included studies [16, 19–25]. The result of the meta-analysis shows that the annual cumulative incidence of hip fractures was 121.39 (95% CI: 82.93–177.69) per 100,000 persons in men and 129.82 (95% CI: 104.90–160.66) per 100,000 persons in women (Fig. 2). The pooled standardized annual cumulative incidence of hip fractures was 138.26 (95% CI: 98.71–193.65) per 100,000 person and 157.52 (95% CI: 124.29–199.64) per 100,000 persons in men and women, respectively (Figs. 3 & 4).

Although there was a lack of information about the participants, meta-regression was used to investigate potential sources of heterogeneity between the studies by including the study characteristics (i.e., year of publication and province). The results did not show any significant relationship (*P*-value = 0.971 for year of publication and *p* = 0.873 for province).

**Fig. 5:**
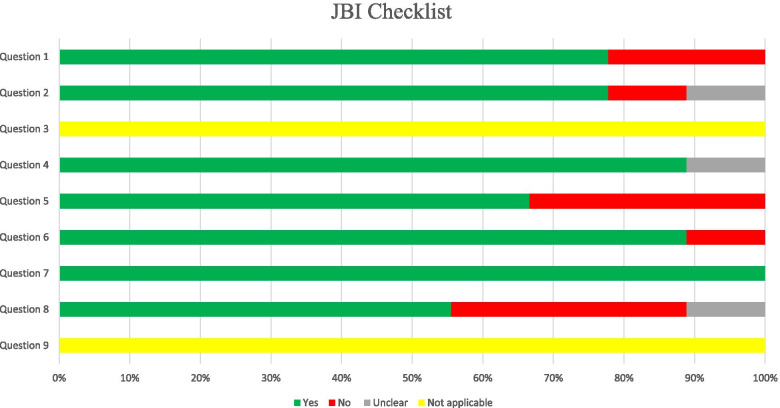
Risk of bias assessments using JBI tool

The results of the Egger’s test showed the possibility of publication bias (*P* < 0.05). The risk of bias was assessed using the nine-question JBI critical appraisal checklist for studies reporting prevalence data (Fig. 5). Every question has four possible answers (i.e., yes, no, unclear and, not applicable). The questions are as follow: 1) Was the sample frame appropriate to address the target population? 2) Were study participants sampled in an appropriate way? 3) Was the sample size adequate? 4) Were the study subjects and the setting described in details? 5) Was the data analysis conducted with sufficient coverage of the identified sample? 6) Were valid methods used for the identification of the condition? 7) Was the condition measured in a standard, reliable way for all participants? 8) Was there appropriate statistical analysis? 9) Was the response rate adequate, and if not, was the low response rate managed appropriately?

## Discussion

This systematic review and meta-analysis identified seven studies reporting osteoporotic fracture incidence rates. The meta-analysis on 4815 participants enabled us to assess reliable incidence rates of osteoporotic fractures in Iran. Results revealed that women had a higher annual cumulative incidence of hip fracture.

The pooled cumulative incidence of hip fractures shows that Iran has intermediate hip fracture rates compared to other Asian countries and global statistics. Previous studies showed several countries in Asia such as Taiwan (with the standardized rates of 233.4 and 496.8 in men and women, respectively) and Hong Kong (with the standardized rates of 193 and 484.3 in men and women, respectively) have a higher incidence rate of hip fracture than Iran.Among the countries in the middle eastern countries, the age-standardized hip fracture rates were reported as 216.6 in men and 316 per 100,000 populations in Kuwait [26] and 180 and 256 in men and women in Lebanon [27].

The incidence rate of osteoporotic fractures is diverse throughout the countries. Some studies showed a significantly lower incidence of hip fracture in black and Asian people [26]. Osteoporotic fracture rates are higher in western countries than in other regions. Despite the lower population, in 2000, almost 34.8% of all osteoporotic fractures in men and women aged over 50 years occurred in Europe, while the corresponding figure in Eastern Mediterranean was 2.9% [28]. The age-standardized hip fracture rates (per 100,000 population) was varied between 137.8 and 346 among men and women in Switzerland to 567 and 759 among men and women in Asteria. These values were reported as 197.2 in men and 553.5 in women in the United States [26]. Besides various reports presented in different countries, some differences were seen in different cities or regions inside a country, as the incidence rate of age-standardized hip fracture among women in Minnesota was reported as 511.5 per 100,000 populations [26].

The results of the present study showed a considerable variation in the incidence rate of osteoporotic fractures throughout the country; such variation was also reported at the international level [28, 29]. Several risk factors that are associated with osteoporotic fractures, such as low BMD, low calcium intake, reduced sunlight exposure, menopause status, smoking status and, physical activity, may have a substantial effect on the variation in the osteoporotic fractures incidence rate [30].

The increasing pattern of pooled incidence rates of hip fractures according to age has been verified in previous studies [6]. The result of an age-specific meta-analysis shows that the growth trend of the incidence of hip fractures in older women is higher than in men. The difference in the pattern of hip fracture incidence in men and women has been verified in previous studies [31]. In Iran, approximately 7.3% of the population were aged > 60 years in 2006, while it was increased to 9.3% in 2016 [32] and it is projected to rise to 31% by 2050, which is higher than the global average [33]. These demographic changes in Iran, like other countries, will lead to the increased medical costs associated with chronic diseases such as osteoporosis [34], the increased rate of mortality, and the lower quality of life in survived patients. So, it is necessary to provide comprehensive interventions to address osteoporotic fractures in a country. A recent systematic review and meta-analysis showed that screening in primary care is an appropriate option to decrease osteoporotic fractures and should be reflected by guideline committees. The authors highlighted that screening programs could prevent osteoporotic fractures, especially hip fractures in older women [35]. Moreover, although the risk of the subsequent fracture is increased following a previous osteoporotic fracture, there is a large care gap, and generally, less than 50% of fractures are being assessed for bone health and linked to the appropriate treatments [36]. To address this gap, Fracture Liaison Service (FLS) has been presented as a practical approach to identify patients with fragility fractures and link them to diagnosis and treatment services as well as fall prevention and osteoporosis-related education for lifestyle modification [36, 37]. The pilot centers for the FLS were recently established in Iran in different cities, and the program will be extended according to the experiences that resulted from the piloting phase. Considering the effectiveness of a fracture liaison service to reduce the re-fracture rate [38, 39], establishing and expanding this program can significantly help reduce the rate of osteoporotic fractures in Iran’s aging population.

This study provided valuable data on the incidence of osteoporotic fractures about which there is a large information gap; however, the results suffer from the following limitations: first, a low number of studies that were eligible for inclusion; second, the lack of population-based studies, which were the most critical limitations of this study; third, the lack of information by fracture site and presence of highly heterogeneous studies despite the use of a random effect model.

## Conclusion

This study showed a considerable incidence rate of osteoporotic hip fractures in Iran. Considering the country’s aging population, diagnosis of high fracture risk individuals in primary care, and reducing re-fractures by establishing fracture liaison services should be considered by policymakers as the evidence-based approaches to reduce osteoporotic fractures in Iran.

## Supplementary Information

**Additional file 1.** Electronic Database Search Strategy.

## Data Availability

The datasets used and/or analyzed during the current study are available from the corresponding author on reasonable request.
