# Supplementary material for: Annual incidence of osteoporotic hip fractures in Iran: a systematic review and meta-analysis
Source: BMC Geriatr. 2021 Nov 30;21:668. doi: 10.1186/s12877-021-02603-1 (PMC8638533; doi:10.1186/s12877-021-02603-1)
Supplement: Supplementary file 1 — Additional file 1. Electronic Database Search Strategy. [file 12877_2021_2603_MOESM1_ESM.docx]

Electronic Database Search Strategy

MEDLINE through PubMed:

1. "Fractures, Bone"[Mesh] OR Fracture*[Tiab] OR Broke*[Tiab]
2. "Incidence"[Mesh] OR ‎incidence[Tiab] OR epidemiolog*[Tiab] OR probabilit*[Tiab] OR "disease frequency"[Tiab] OR occuren*[Tiab] OR risk[Tiab] OR rate[Tiab] OR prevalence[Tiab]
3. “Iran"[Mesh] OR Iran[All Fields] OR "Islamic Republic of Iran"[All Fields] OR "IRIran"[All Fields] OR "I R. Iran"[All Fields] OR Persia*[All Fields]
4. #1 AND #2 AND #3
5. Animal*[Tiab] OR mice[Tiab] OR mus[Tiab] OR rat[Tiab] OR mouse[Tiab]
6. #4 NOT #5

Embase (via Elsevier):

1. exp Fractures, Bone / OR Fracture*:ab,ti OR Broke*:ab,ti
2. exp Incidence/ OR ‎incidence:ab,ti OR epidemiolog*:ab,ti OR probabilit*:ab,ti OR "disease frequency":ab,ti OR occuren*:ab,ti OR risk:ab,ti OR rate:ab,ti OR prevalence:ab,ti
3. exp Iran/ OR Iran OR "Islamic Republic of Iran" OR "IRIran” OR "I R. Iran" OR Persia*
4. #1 AND #2 AND #3
5. Animal*:ab,ti OR mice:ab,ti OR mus:ab,ti OR rat:ab,ti OR mouse:ab,ti
6. #4 NOT #5

Scopus:

1. TITLE-ABS-KEY ( fracture OR broke* )
2. TITLE-ABS-KEY ( epidemiolog* OR ‎incidence OR probabilit* OR "disease ‎frequency" OR occuren* OR risk OR rate OR prevalence )
3. Iran OR "Islamic Republic of Iran" OR "IRIran" OR "I R. Iran" OR Persia*
4. #1 AND #2 AND #3
5. TITLE-ABS-KEY ( animal* OR mice OR mus OR rat OR mouse )
6. #4 NOT #5

Web of Science (core collection; Clarivate):

1. TOPIC: ( fracture OR broke* )
2. TOPIC: ( epidemiolog* OR ‎incidence OR probabilit* OR "disease ‎frequency" OR occuren* OR risk OR rate OR prevalence )
3. Iran OR "Islamic Republic of Iran" OR "IRIran" OR "I R. Iran" OR Persia*
4. #1 AND #2 AND #3
5. TOPIC: ( animal* OR mice OR mus OR rat OR mouse )
6. #4 NOT #5
